# Ethical reflections in legal medicine and forensic medical expertise: looking beyond the norm

**DOI:** 10.47626/1679-4435-2025-1478

**Published:** 2025-09-22

**Authors:** Gustavo de Almeida

**Affiliations:** 1 Serviço de Qualidade de Vida e Saúde Ocupacional, Senado Federal, Brasília, DF, Brazil

**Keywords:** bioethics, medical ethics, forensic medicine, physician-patient relations, social justice., bioética, ética médica, justiça social, medicina legal, relações médico-paciente.

## Abstract

Forensic medical expertise, as an expression of legal medicine, is ethically distinct from the traditional Hippocratic model of clinical care. Its moral framework must be adapted to the specific purpose of informing administrative, legal, and governmental decisions, thereby contributing to social equity. Drawing on both printed literature and digital database research, this article reflects on the dynamics of the physician-patient relationship - i.e., between the medical examiner and the examinee - and the role of the expert within the context of social justice. It argues that the expert’s conduct should extend beyond a strictly normative deontology or a pragmatic utilitarian approach and be also guided by the virtues of character. In doing so, forensic experts can uphold both the quality standards required by modern medicine and the enduring values of the medical profession.

## Abstract (pt)

Como expressão da atividade médica legal, a perícia médica distingue-se eticamente da prática assistencial de tradição hipocrática, exigindo que a sua moralidade seja devidamente ajustada ao objetivo de auxiliar decisões administrativas, legais e estatais, contribuindo para a promoção da equidade social. Este artigo, baseado em literatura física e em pesquisas em bases digitais, visa refletir sobre a relação entre médico e paciente - neste caso, entre perito e periciado - e a função desse especialista no contexto da justiça social. Percebe-se que os ditames de conduta do perito devem avançar para além de uma deontologia puramente normativa ou de um utilitarismo pragmático, recebendo também a orientação das virtudes do caráter. Assim, são atendidos tanto os padrões de qualidade exigidos pela medicina atual quanto os valores seculares da profissão.

## INTRODUCTION

Forensic medical expertise, here used synonymously with legal medicine, differs from traditional therapeutic practice. Its focus on health is indirect as it seeks to apply knowledge to other fields - especially legal domains such as criminal, civil, administrative, social security, and insurance law. As such, forensic medical expertise plays a critical role in decision-making within administrative, law enforcement, judicial, and health policy contexts.

From the perspectives of teleology, deontology and rights-based ethics, this article addresses ethical elements of forensic medical activity and its role in supporting decisions in the legal and social spheres.

## DISCUSSION

### CARE *VS*. EXPERTISE

One of William Osler’s best-known aphorisms states that “medical practice is an art based on science,”^1^ where practitioners are able to apply broad technical knowledge to individual cases - a blend of “episteme” and *phronesis*, in the language of Greek philosophy.^2^ Furthermore, the field of medicine, shaped by historical and cultural values and influences, carries with it a moral epistemology.^3^

To define best professional practices, medical codes of ethics are eminently deontological (i.e., based on duties of conduct). They apply to all areas of medicine, though they often focus more on patient care, in line with the Hippocratic tradition. On the other hand, forensic medical expertise is a branch of medicine aimed not at providing care, but at answering questions that arise from other domains of human activity.

In fact, until the mid-19th century, medical expert work revolved around social medicine, which was closely linked to the hygienist movement. The growing adoption of the scientific method, alongside the uneven progress of legal systems, made legal medicine a key tool in several fields of law, including criminal, civil, administrative, labor, social security, and insurance law.^4^ In a broad sense, medical expertise, legal medicine, and forensic medicine are thus interchangeable terms.

Despite following the rites of the legal system, forensic medical expertise avoids procedures that would only seem to meet procedural formalities without reflecting the actual truth of the facts - an approach that could lead to misleading interpretations over the course of the expert’s work.^5^

The words of Nerio Rojas, the renowned Argentinian forensic psychiatrist, are indeed still relevant: “The duty of an expert is to tell the truth, but one must first know how to find it and then be willing to speak it: the former being a scientific matter; the latter, a moral one.”^4^

Given these particularities, the creation of a specific ethical canon for legal medicine has been considered. However, there is no record of such a canon having been implemented in any country, although numerous guidelines exist for forensic medical practice.

### THE PHYSICIAN-PATIENT RELATIONSHIP

Medicine is a unique profession, valued for its ability to relieve human suffering.

Manifestos such as the Hippocratic Oath and the Declaration of Geneva underline the importance of loyalty to the patient. Nevertheless, even when a physician commits to caring for a patient in a “Kantian” sense - i.e., treating them as an end in itself and not merely as a means - the relationship between the two is marked by clear asymmetry. The physician holds technical knowledge, while the patient passively “endures” such expertise, as implied - etymologically or not - by the very word “patient.”^2^

The introduction of informed consent requirements represents a step forward, but there is still a certain power imbalance, as consent does not erase the patient’s sense of estrangement when in the medical environment.^6^,^7^

### THE EXAMINER-EXAMINEE RELATIONSHIP: MAXIMUM IMBALANCE

The examiner diverges from the traditional archetype of the physician, the one shaped by the *animus* of beneficence. Bound by impersonality, the examiner’s decisions do not seek to please the examinee, who is, in turn, not entitled to choose their examiner.^8^

Although there has been a movement toward empowering patients in clinical care settings, this shift has not been reflected in the medicolegal sphere.^9^ In this context, the examinee has limited autonomy, and the lack of follow-up consultations makes it difficult to build trust between patient and physician. This dynamic increases the imbalance in the relationship, as seen in clinical practice and as discussed in certain models of Game Theory.^10^ It is therefore essential to ensure that this imbalance does not turn into vulnerability, which could undermine the examinee’s dignity.^11^

Even when examiners suspect symptoms might be fabricated (malingering), they should remain polite and respectful. Likewise, confidentiality should only be broken as much as needed to fulfill professional duties while respecting the limits set by different jurisdictions.^12^

### SOCIAL JUSTICE AND FORENSIC MEDICAL EXPERTISE

Justice is a multifaceted concept with many historical references. It appears in the retributive principle of the Law of Talion (Babylonian Code of Hammurabi; Old Testament); in Aristotle’s idea of virtue centered on the common good; in Bentham and Mill’s pragmatic utilitarianism; in Kant’s coexistence of freedoms; in Hobbes, Locke, and Rousseau’s contractualism and social peace; and in Kelsen’s correspondence between legality and normative order.^4^

Indeed, justice is viscerally linked to social coexistence, as illustrated by the myth of Protagoras: although humans learned techniques such as the use of fire (gifted by Prometheus), they failed to form communities, for they kept committing atrocities against each other. Scattered, they remained vulnerable to wild animals, enemies, and the elements. This highlights the political and civilizing predicate of justice and solidifies the State’s role in fostering harmonious coexistence and the protection of individuals.^13^

The State must therefore demonstrate qualities that validate its actions (including coercive power), whose moral legitimacy involves treating citizens equally.

Nevertheless, social justice does not demand absolute equality, i.e., being insensitive to natural differences between individuals, groups, and communities. In this regard, Rawls’ “veil of ignorance” is a useful allegory for ensuring fair distribution of resources and opportunities and enabling the Welfare State.^14^

Similarly, the bioethical principle of justice guides many medical decisions and is especially relevant for forensic medicine. Here the expert acts as a gatekeeper for access and benefits, balancing loyalty between their government role and the person being assessed, whose autonomy may need to be limited in the name of collective and institutional interests.

This constant shift between Aesculapian paternalism and the impartiality of a judge can cause psychological strain for the expert, who paradoxically fulfills their social responsibility duties through strict professional, technical and ethical rigor.^15^

### VIRTUE AS THE FOUNDATION FOR BEST MEDICAL PRACTICES

Even though forensic medical practice recognizes the existence of ethical and moral nuances in individual cases, it is often based on utilitarianism and its collectivist bias.^8^ A more balanced approach would draw from different theories, such as a not merely normative form of deontology (ethics of duty) and aretology (ethics of virtue).

With respect to the latter point, virtue is built through education, experience, and habits, leading to a dynamic that pushes the expert toward excellence. Pellegrino and Thomasma, based on MacIntyre’s thinking, highlight eight essential virtues in their classical essay “The Virtues in Medical Practice”: trust, compassion, prudence, justice, courage, temperance, and effacement of self-interest.^16^

For individual cases, the dominant approach based on Beauchamp and Childress’ principlism seems limited. Such instances call for character-based conduct, especially when dealing with patients who are more vulnerable to external influences. In these situations, both *erga omnes* imperatives and excessive consequentialism may not be appropriate for decision-making.^17^,^18^

Medicine is a combination of science, art, and morality. Indeed, a forensic medical expert’s ethical system should reflect personal convictions (autonomy), not fear of sanctions (heteronomy).^3^ To achieve this, Foucault’s concept of “care of the self”, drawn from Antiquity, is useful here. Within this precept, the physician engages in reflective self-examination and processes conflicts consciously. With a clear identity, the expert works spontaneously and in flow, free from the burden intrinsically imposed by their sanitary authority - the “biopower,” as described by the French philosopher.^19^

In the eudaimonic pursuit of virtue, being and duty are inseparable. This honors both the origins of the profession and the high standards required by modern medicine.^18^,^20^

### DIALECTICS AS A TOOL FOR BEST MEDICAL PRACTICES

Despite technological advances, healthcare practices have not progressed at the same pace. The most likely reason for this is the widely reported difficulty in communication between physicians and patients.

Often there is a vertical dialogic structure where the physician speaks and the patient listens while setting aside their subjectivity. To avoid reducing the relationship to a mere clinical case, mutual understanding must be sought. This is especially important in today’s pluralistic society, where the constant is, paradoxically, a high variability in views and values. Making interactions more horizontal means allowing the patient to take part in their own narrative, as well as reducing the “moral strangeness” as described by Engelhardt.^3^,^18^

In the context of the medicolegal field, it may not be reasonable to expect empathy from the expert. However, it is through exercising alterity that the examiner allows the examinee to share their own context and bring out their personal history and unique worldview. These elements make the technical analysis stronger.

Such prudent conduct acknowledges the limits of human knowledge and the inevitable fallibility of medical science while still ensuring best practices through proper and timely decision-making (*recta ratio agibilum*).^11^

### LONG COVID AND WORK DISABILITY

In light of the above reflections, it is relevant to examine the work capacity of people affected by post-COVID syndrome, which represents a growing public health issue with a substantial global disease burden.^21^

Long COVID, more common in unvaccinated adults, refers to a range of symptoms that appear during or after an acute infection and persist for more than three months. Common symptoms include brain fog, fatigue, shortness of breath, chest pain, palpitations, and headaches. Because clinical and laboratory findings are limited, the lack of objective evidence creates tension between the patient’s perception and the medical assessment. Without organic proof, these cases are often labeled as “medically unexplained symptoms” or functional syndromes.^22^

It is also difficult to establish a clear link between the reported symptoms and a SARS-CoV-2 infection. This further complicates the already challenging process of determining causality.^23^

Such uncertainty can lead to mutual dissatisfaction between physicians and patients, especially when patients request work disability certificates or medical leave.^15^

Considering that prolonged absence from work affects not only a person’s financial situation but also their psychological well-being, it is essential to identify obstacles to returning to work as early as possible. The framework of the International Classification of Functioning, Disability and Health (ICF) offers useful concepts for this, such as barriers, contextualized performance, and participation.^24^

As a complement, the American Medical Association (AMA) framework for assessing work fitness considers three dimensions: risk (probability of self-harm or harm to others), capacity (psychophysical ability to perform tasks), and tolerance (willingness to cope with symptoms in exchange for the benefits of working). Tolerance, due to its subjective nature, may influence and strengthen the disability assessment, but does not determine the outcome.^25^

The return-to-work process should be gradual and tailored to the patient’s needs. Recommended strategies include detailed occupational assessments, symptom monitoring, and workplace adjustments such as flexible schedules or phased returns.^26^ These measures reduce psychophysical strain and provide a sense of purpose and self-esteem.^27^

In this scenario, and given the uncertainty around long-term effects of COVID-19, physicians should take a cautious approach with patients in the workforce while following the *primum non nocere* (do no harm) maxim. At the collective level, the bioethical principle of precaution should guide return-to-work policies, ensuring job stability and reasonable accommodations.^28^

Finally, it is essential to acknowledge that research on long COVID is still evolving. This reflects the intellectual humility expressed in the aphorism *ars longa, vita brevis -* a timeless piece of wisdom that can be traced back to Hippocrates, the Stoics, and, more recently, to the insights of Russell, Dunning, and Kahneman.^29^,^30^

## CONCLUSIONS

Medicine is a combination of science, art, and morality.

Due to its legal impact, forensic medical expertise requires both formalism and impartiality - qualities that set it apart from traditional healthcare practices. The ethical values and principles that guide this activity must therefore be shaped in light of its specific objectives.

Unlike in the Hippocratic model of compassion, in forensic medical work, recognizing the power imbalance is an expression of the alterity cultivated by a virtuous expert. This approach is in line with the self-effacement virtue as proposed by Pellegrino & Thomasma: an ego-free stance in which the expert, while maintaining technical rigor, does not ignore the subjective aspects of the other, and seeks to consider them whenever possible.

The expert’s ethics must reflect their own character and convictions instead of being driven by fear of criticism, dissatisfaction, or punishment. It is a matter of deliberating with prudence - a goal that is only attainable through improvement efforts, both personal and professional.

Utopian or not, it seems hard to deny that excellence carries within it the drive to go beyond ordinary action, i.e., beyond what is merely required as a basic standard of conduct. Perhaps the greatest beauty of medicine - whether preventive, clinical, or forensic - lies in cultivating this virtue of supererogation and in maintaining a lifelong commitment to learning.
